# Application of Digital Image Correlation in Space and Frequency Domains to Deformation Analysis of Polymer Film

**DOI:** 10.3390/ma15051842

**Published:** 2022-03-01

**Authors:** Caroline Kopfler, Sanichiro Yoshida, Anup Ghimire

**Affiliations:** Department of Chemistry & Physics, Southeastern Louisiana University, Hammond, LA 70402, USA; caroline.kopfler@selu.edu (C.K.); anup.ghimire@selu.edu (A.G.)

**Keywords:** linear low-density polyethylene, digital image correlation, optical non-destructive testing, speckle, fourier scaling theorem, gaussian filtering, optical methods

## Abstract

Using speckle patterns formed by an expanded and collimated He-Ne laser beam, we apply DIC (Digital Image Correlation) methods to estimate the deformation of LLDPE (linear low-density polyethylene) film. The laser beam was transmitted through the film specimen while a tensile machine applied a load to the specimen vertically. The transmitted laser light was projected on a screen, and the resultant image was captured by a digital camera. The captured image was analyzed both in space and frequency domains. For the space-domain analysis, the random speckle pattern was used to register the local displacement due to the deformation. For the frequency-domain analysis, the diffraction-like pattern, due to the horizontally-running, periodic groove-like structure of the film was used to characterize the overall deformation along vertical columns of analysis. It has been found that when the deformation is small and uniform, the conventional space domain analysis is applicable to the entire film specimen. However, once the deformation loses the spatial uniformity, the space-domain analysis falls short if applied to the entire specimen. The application of DIC to local (windowed) regions is still useful but time consuming. In the non-uniform situation, the frequency-domain analysis is found capable of revealing average deformation along each column of analysis.

## 1. Introduction

Commercial shipping facilities commonly use linear low-density polyethylene (LLDPE) for packaging freight. For efficiency, wrapping machines are responsible for packaging shipments by rotating thin LLDPE film around the objects. These machines perform this action as quickly as possible to optimize both time and cost of the process. At increased wrapping rates, however, LLDPE film stretches more, ultimately leading to its failure.

**Figure 1:**
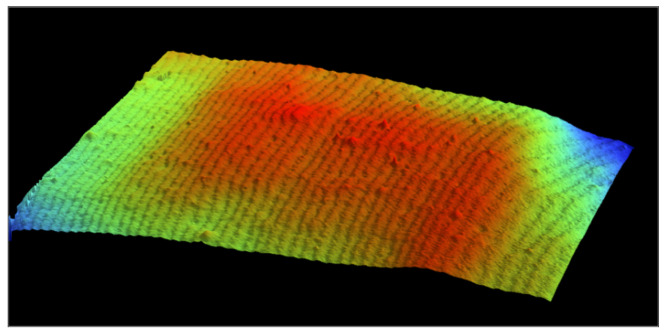
Surface structure of LLDPE film.

The unique characteristics of this film make the material advantageous and multi-functional across industries. LLDPE is a thermoplastic polymer characterized by a predominantly linear backbone and a high proportion of short branches. Due to the material’s structure, it cannot be packed tightly, which gives it a hazy, transparent appearance. In comparison to traditional low-density polyethylene, LLPDE has a crystalline structure with little to no elastic memory or recovery, resulting in increased tensile strength and elongation [1]. During manufacturing, the LLDPE molecules align with the spooling direction to create striations on the film. Up close, these striations present themselves as periodic grooves on the film’s surface, seen below in Figure 1. These grooves cause non-uniform surface height variation, which complicates conventional optical methods based on speckles. In this study, we explore a way to use the structural pattern for optical-base deformation analysis.

While many non-destructive optical testing methods are available to evaluate material deformation [2,3], speckle techniques are most attractive for analysis of LLDPE film. When coherent light is reflected on a rough surface or transmitted through an inhomogeneous medium, the optical field of an imaging device forms a pattern consisting of many bright and dark spots. These spots, known as speckles, result from the coherent superposition of light rays scattered by the medium. Because the light rays reaching a particular spot on the image plane follow optical paths defined specifically by the corresponding section of the medium, the speckle field reveals the specimen’s fingerprints at a given time and position. By analyzing a speckle field formed on the image plane, we can probe the spatiotemporal behavior of the medium, such as the displacement or velocity of the scattering particles.

Traditionally, the subtraction method used in speckle pattern studies [2,3] retrieves displacement through phase analysis of the speckles. Digital Image Correlation [4,5,6] detects the displacement by evaluating the correlation between the speckle fields of the original and deformed states. By relating the spatiotemporal behavior within a dynamic model, we can characterize the material’s deformation properties, such as elasticity and viscosity. These dynamic models are often used to investigate biological materials [7,8,9], which are generally within systems experiencing Brownian motion [10]. While the technique in this study is similar to those used to mechanically characterize dynamic physiological systems, it is important to note that we focus on a quasi-static system, where factors like speckle decorrelation time are negligible. Additionally, the film’s surface height variation presents a problem when optically evaluating change in thickness. This observation motivates exploration of alternative methods for evaluating surface structure and deformation of the material.

The present study applies conventional DIC methods [4,11] using the speckle field generated by the laser beam transmitted through the film specimen, unlike painted speckles often used in similar deformation analysis [12]. The coexistence of scattering-induced speckles and periodic grooves, however, complicates analysis due to the formation of diffraction-like patterns superposed onto the scattering-induced speckles on a projected image. While this image looks like typical patterns from diffraction grating, the interval between neighboring grooves is orders of magnitude greater than the optical wavelength. Therefore, we interpret the pattern on the screen as the result of destructive interference between multiple rays and refer to it as the linear dark-fringe-like interference pattern, simple referred to as linear dark-fringes.

We find that we can use the periodicity of the grooves to estimate the film’s deformation. In the frequency domain, this quasi-constant interval produces fairly sharp peaks in the Fourier spectrum. As the film stretches, the interval between linear dark-fringes increases, which shifts the peak frequency on the Fourier spectrum. From this shift, we can estimate the amount of stretch [13,14]. Here, random speckles superposed to linear dark-fringes compromises accuracy of the analysis [15]. We find that proper Gaussian pre-filtering and selection of frequency range can improve overall accuracy of the analysis [16]. The effectiveness of this method is discussed in the following sections, as well as space-domain analysis using DIC methodology for comparison [4,5,14].

## 2. Materials and Methods

### 2.1. Structural Inhomogenity in LLDPE Film

**Figure 2:**
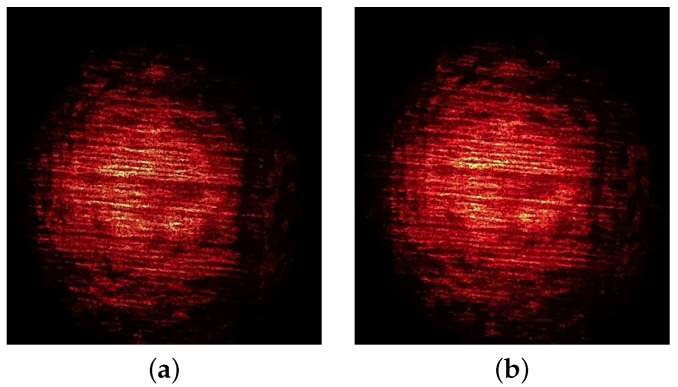
Sample digital images collected during the experiment. (**a**) Original (un-stretched) Image, (**b**) Stretched Image.

The linear backbone and short branches of the LLDPE molecules align in the direction of spooling during the extrusion manufacturing process, resulting in periodic structural grooves observable on the film’s surface, as illustrated in Figure 1. When a light source passes through the transparent film, a projection of the dark fringes and speckle patterns are visible. Figure 2a shows a sample optical pattern projected on a screen.

When an external load stretches the film, the linear dark-fringes and speckles change independently. Figure 2b shows the projected optical pattern after an external load stretches the film sample vertically. Careful examination of Figure 2b will reveal that the interval of the horizontally running linear dark-fringes increases from Figure 2a and that speckle patterns expand vertically.

Recall, Figure 1 shows the image formed by a surface profiler (Bruker Contour GT-KO, courtesy of University of New Orleans). Vision-64 Analysis Software indicates approximately 40 peaks along the y-axis over a span of 25 mm. This indicates that the pitch of the periodic structure is 25 mm/40 ≈ 600 µm, which is three orders of magnitude greater than the laser’s wavelength of 632.8 nm. It is unlikely that this periodic structure forms diffraction patterns similar to those formed by diffraction grating. However, the groove-like structure causes the transmitted light to form a periodic pattern of destructive interference that resembles a diffraction pattern.

The random nature of speckle patterns on the projected image makes application of traditional optical interferometric techniques difficult based on the phase of the source-light such as holographic inteferometry [15,17]. Speckle-pattern interferometry is applicable, but linear dark-fringes compromise the accuracy because the spatial shifts of speckles and linear dark-fringes are different from each other. The speckle-pattern shift results from physical displacement of the local area, whereas the shift of the linear dark-fringes results from the deformation of structural grooves. When changes are small and uniform, the two types of shift may represent the same deformation; however, when deformation is non-uniform, the shifts are likely to behave differently.

### 2.2. Experimental Setup

The experimental setup used for data collection consisted of several parts. Initially, several LLDPE film samples with thicknesses of 10 µm and 20 µm were cut into 4 cm squares and attached to an ADMET tensile machine’s stationary and dynamic grips. Recall that the film’s surface structure contains grooves in line with its spooling direction. Thus, in separate experiments, the samples were oriented so that these striations were perpendicular (horizontal) and parallel to the pulling direction (vertical).

**Figure 3:**
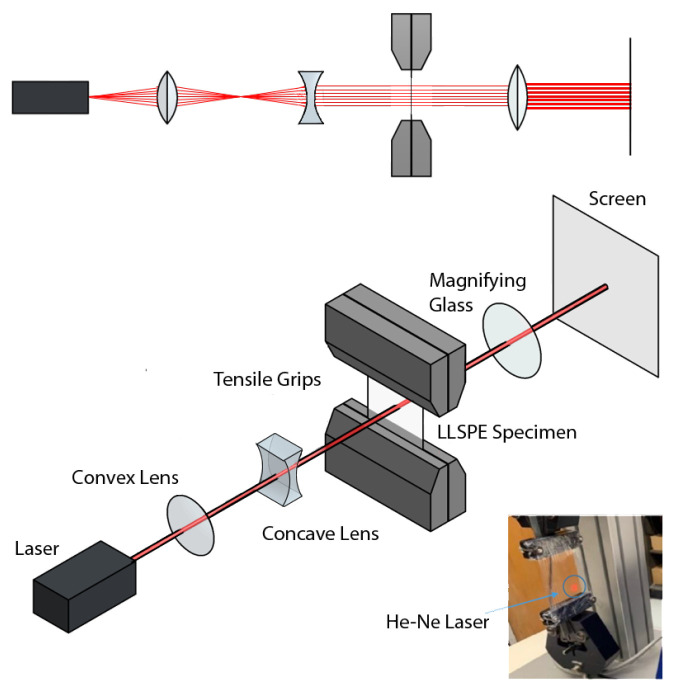
Experimental setup, showing the laser passing through the specimen.

A 17 mW Helium-Neon laser provided the light source for experiments. The laser beam expands as it propagates, and collimation is necessary for obtaining accurate interferometric patterns. Initially, we suspected that polarization could affect the film’s transmission characteristics, which was investigated by varying the polarization. We did not find a noticeable effect of polarization, which lead us to the experimental setup shown in Figure 3.

Additionally, the spot size of the beam is increased by a factor of 10. The beam expander consists of two lenses with focal lengths of 25 mm and 250 mm. The radius of the laser beam on the specimen is 5 mm and covers approximately 20% of the film’s surface. Additionally, the beam’s waist size is 0.5 mm. Multiple studies including beam spot size measurements along the propagation axis and cross-sectional beam profile measurements using a charge-coupled device camera verified that the laser beam is in the Gaussian mode (TEM00) [18]. We adjusted the ƒ# of the imaging lens to optimize speckle size [19,20].

Digital images were taken as the tensile machine pulled the specimen. The digital camera was set to take images as the material stretched in elongation intervals of 50 µm. The image correlation procedure, described in the next section, compares pairs of images to evaluate the corresponding deformation. The camera specifications follow in Table 1.

### 2.3. Principles of Operation

#### 2.3.1. One-Dimensional DIC Method in Space-Domain

The following method finds the average stretch, or normal strain, over a column (or row) of a projected image. Here, the normal strain is defined by Equation (1), where dl is the elongation (compression) and *l* is the initial length [13].

(1) $$\epsilon =\frac{dl}{l}$$

The four steps below describe the analytical process for 1D analysis in the space-domain.

**Figure 4:**
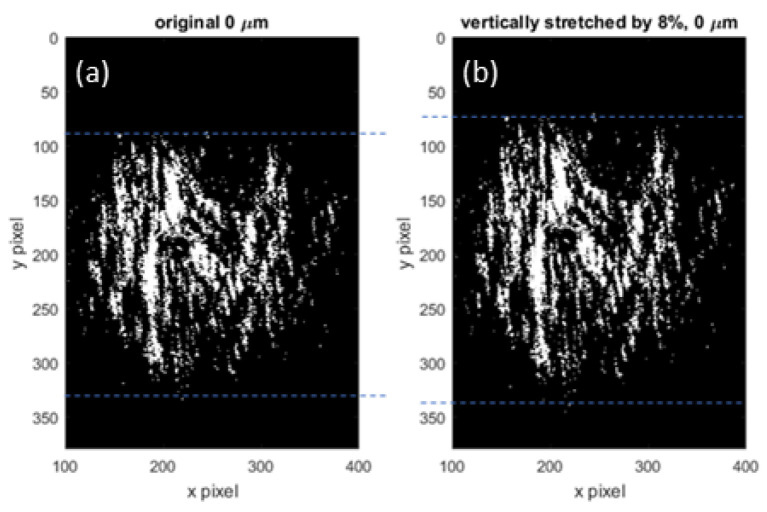
Original image (**a**) and artificially stretched by 8% (**b**).

1. Stretch the original, un-stretched image by artificially stretching the axis for a given scaling factor. Figure 4 exhibits an example of an original sample image (a) stretched numerically along the vertical axis by a factor of 8% (b).
2. Compare the physically stretched image with the artificially stretched image by computing the correlation coefficient $C_{cor}$ defined by Equation (2) over a column for a vertical stretch or a row for a horizontal stretch.
  (2) $$C_{cor}=\frac{cov(a,b)}{\sqrt{cov(a,a)cov(b,b)}}$$
  The variables *a* and *b* are the column (row) vectors containing gray-scale values of un-stretched and stretched image. In Equation (3), cov(a,b) is the covariance of vectors *a* and *b* [21].
  (3) $$cov\left(a,b\right)=\frac{\sum_{i=1}^{n}\left(a_{i}-\bar{a}\right)\left(b_{i}-\bar{b}\right)}{n-1}$$
  Here, $\bar{a}$ and $\bar{b}$ are the mean values of the respective vectors’ elements.
3. Repeat 1 using a different stretching factor and compute the cross-correlation. Iterate this procedure to find the maximum cross-correlation. Determine the stretch of the selected column (row) as the stretch factor that maximizes the cross-correlation.
4. Repeat 1–3 for all the columns (rows) to find the average stretch (normal strain) for each column (row).

#### 2.3.2. Two-Dimensional DIC Method in Space-Domain

**Figure 5:**
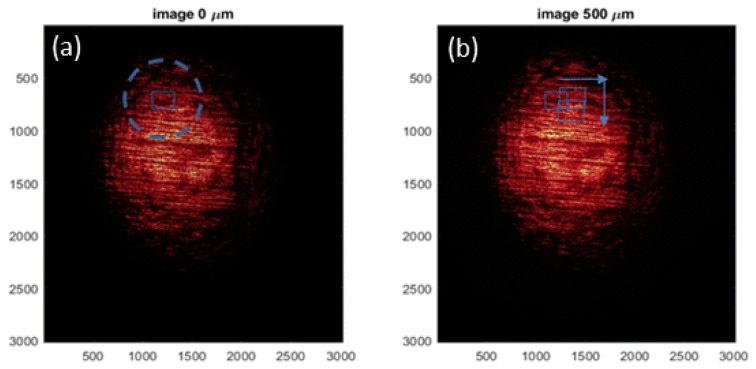
A windowed pattern chosen as the kernel to compute the correlation between the two images.

This method is generally known as the convolutional DIC technique [22]. A small window called a kernel is set up in the original (un-stretched) image, as shown in Figure 5a. Kernels are groups of elements within an image. An element represents a pixel value that corresponds to the target position within the kernel. In the stretched image, the DIC algorithm moves the kernel vertically and horizontally on a pixel-by-pixel basis and computes the correlation using Equation (4) at each coordinate point [23]. Here in Equation (4), $p_{x}$ represents the movement of the kernel, where the first function f(x) represents the gray-scale pixel value at coordinate *x* before the stretch and g(x) is the gray-scale pixel value at *x* after the stretch.

(4) $$\left(f★g\right)\left(p_{x}\right)=\int_{-\infty }^{\infty }f\left(\tau \right)g\left(p_{x}+\tau \right)d\tau$$

The left-hand side of Equation (4) indicates that the cross-correlation is a function of kernel movement $p_{x}$. In the same fashion, the kernel moves along the *y*-axis by $p_{y}$, and the pair of $p_{x}$ and $p_{y}$ that maximizes the cross-correlation is recorded. The pair $\left(p_{x},p_{y}\right)$ constitutes the displacement vector as the center pixel of the kernel [23]. By examining all the area of interest in this way, we can estimate the local displacement vector.

For clarification, the DIC process using Equation (4) in a numerical method calculates the similarity between two signals. In the context of this study, the two images serve as the *input* and *output* signals, respectively, where the input function is the image before elongation, and the output function is the image after elongation.

#### 2.3.3. Frequency Domain Method

The one-dimensional DIC method discussed above uses the speckle pattern along the entire length of a column of interest. This method works well when the speckle patterns change in the same fashion as the artificial elongation, which assumes uniform stretch. In reality, the deformation of the film specimen becomes non-uniform at a low level of elongation. This situation makes it difficult to apply the one-dimensional DIC method to a realistic situation where deformation of the wrapping film undergoes non-uniform stretching. The convolutional DIC method is applicable in this situation; however, it is unrealistic and time-consuming.

The frequency domain method solves the problem by utilizing differentiation and scaling properties of the Fourier transform. When deformation of the film specimen is not uniform at the local level so that the one-dimensional space domain method is not applicable, it is often the case that the deformation is uniform enough at the global scale. The periodic groove-like structures can serve as a gauge to evaluate overall deformation. Under this condition, the periodicity of dark-fringes due to the groove-like structures form a quasi-single peak in the frequency domain. As the film stretches, the peak shifts on the frequency axis, which can be interpreted as scaling in the space domain. In this situation, we can estimate the scaling factor in the frequency domain as follows.

Let function f(x) be representing the gray-scale variation of the speckles along the *x*-axis, ξ be the new axis after the compressing/stretching the original axis by a factor of α (ξ=αx), g(ξ) be the derivative of f(ξ) with respect to ξ ( g(ξ)=df(ξ)/dξ), and ω be the angular frequency associated with the Fourier transform of g(ξ). From the differential and scaling properties of Fourier transform, we can obtain the following equation that relates the Fourier transform of g(ξ), $G_{\alpha }\left(\omega \right)$, and axis scaling factor α.

(5) $$G_{\alpha }\left(\omega \right)=\left|F\left\{\frac{df(\xi )}{d\xi }\right\}\right|=\frac{\omega }{\alpha }G\left(\frac{\omega }{\alpha }\right)$$

Here, F denotes the Fourier transform operation. Appendix A describes this logic in detail, including the derivation of Equation (5).

**Figure A1:**
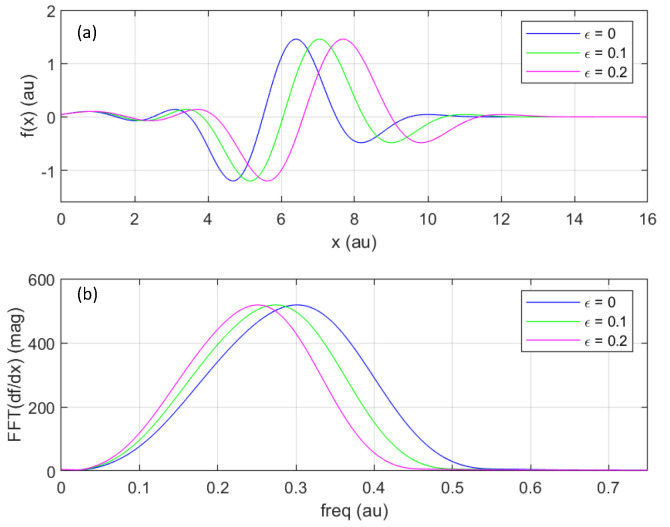
(**a**) Sample function with stretching, (**b**) Fourier transforms of functions in (**a**).

Equation (5) indicates that $G_{\alpha }\left(\omega \right)$ takes the same form on the frequency axis regardless of the value of α; at the peak frequency ${\left(\omega /\alpha \right)}_{peak}\equiv \phi_{peak}$, $G_{\alpha }\left(\omega \right)=\phi_{peak}G\left(\phi_{peak}\right)$, at $\left(\omega /\alpha \right)=\phi_{0}$, $G_{\alpha }\left(\omega \right)=\phi_{0}G\left(\phi_{0}\right)$, .... If the area is enclosed by $G_{\alpha }\left(\omega \right)$ and the frequency axis (see the simplified illustration in Figure A1), it is proportional to 1/α. Thus, comparing this area at scaling factor α with its corresponding area in the un-stretched situation, we can find α.

#### 2.3.4. Analytical Steps

We took the following steps to implement the above algorithm.

1. Gaussian filter the optical image projected on the screen. In this algorithm, the local speckles due to the scattering of the film material become noise while the periodic pattern of the linear dark-fringes produces the signal. Since the local speckle pattern has higher spatial frequency than the linear dark-fringes, low-pass filter the optical image to increase the signal-to-noise ratio.
2. Numericaly differentiate the gray-scale value of the original image with respect to the coordinate variable that is set parallel to the tensile axis. This step is to evaluate df(ξ)/dξ term in Equation (5).
3. Take the FFT of the differentiated gray-scale obtained in step 2. Call this resultant spectrum the original Fourier spectrum. Numerically integrate the original Fourier spectrum for a selected spatial frequency range. This step is to evaluate the area enclosed by the Fourier spectrum and the frequency axis. Call the resultant value the original spectrum-frequency area. The frequency range for this integration should contain the spectral peak and exclude the high frequency region removed by the Gaussian filter. Since the optical intensity varies at each image captured for various reasons such as the change in the background optical intensity, normalize the spectrum-frequency area for the selected frequency range by dividing it by the Fourier spectrum-area of the entire frequency range.
4. Repeat steps 1–3 after the specimen stretches to the current elongation. This process yields the Fourier spectrum for a given stretch factor and the corresponding (current) spectrum-frequency area. Call the resultant spectrum-frequency area the current normalized spectrum-frequency area. Iterate this step for other stretch factors by further elongating the specimen. This procedure yields multiple current normalized spectrum-frequency areas.
5. Compare the current normalized spectrum-frequency areas obtained in step 4 with the original normalized spectrum-frequency area. From the ratio of the current spectrum-frequency area to the original spectrum-frequency area, determine the axis compression factor α. From the axis compression factor, evaluate the stretching factor as ϵ=1/α.

We present the actual process of the above steps in Section 3.3.

## 3. Results and Discussion

### 3.1. Visible Estimation of Displacement Due to Stretch

**Figure 6:**
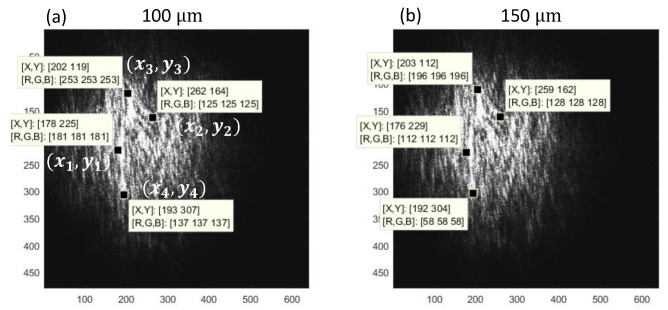
Four reference points used for visual estimation of displacement.

Prior to the application of the above method, we estimate the horizontal and vertical displacement of the image Figure 4a when the tensile load elongates the specimen by 50 µm. For this estimation we select four coordinate points (call the reference points) in the image where the intensity patterns are distinctive. Figure 6a shows the four reference points $\left(x_{1},y_{1}\right)-\left(x_{4},y_{4}\right)$ when the specimen is elongated by 100 µm from the un-stretched state. Figure 6b indicates the coordinates of these distinctive intensity patterns when the specimen is elongated by an additional 50 µm.

The difference in the coordinates of each reference point gives us the displacement vector. Table 2 and Table 3 list the change in these coordinates before and after the additional elongation of 50 µm. Here the former table is for the horizontal displacement and the latter table is for the vertical displacement.

From Table 2 and Table 3, we can estimate the horizontal and vertical strain caused by the additional 50 µm elongation to be 2.0% (horizontal compression) and 2.2 (vertical stretch). Notice that the magnitude of the horizontal and vertical strain are mutually similar, not reflecting Poisson’s ratio of LLDPE (approximately 0.4) [1], indicating that the deformation is not elastic. In the following section, we evaluate the results of the space-domain DIC methods described above referring to the data shown in Table 2 and Table 3.

### 3.2. Digital Image Correlation Method in Space-Domain

#### 3.2.1. One-Dimensional DIC

**Figure 7:**
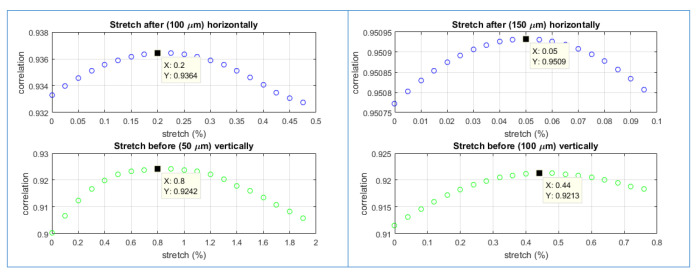
Results from one-dimensional image correlation method in space-domain.

Figure 7 shows the correlation data from the one-dimensional DIC method. Here, the correlation coefficient based on Equation (2) is plotted against the stretching factor ϵ for artificial elongations of 50 µm to 100 µm, and 100 µm to 150 µm (from the left to right). The upper graphs are for the horizontal strain and the lower graphs are for the vertical strain. Since the specimen stretches vertically, the horizontal strain is compressive and the vertical strain is tensile, according to Poisson’s effect. The data shown in Figure 7 is along row 200 in Figure 6a for the horizontal cases and column 200 for the vertical cases.

The correlation data in Figure 7 under each condition show a peak value. The artificial stretching factor corresponding to a peak (called the peak stretching factor) indicates when the intensity pattern of the original image is stretched for this amount and the intensity profile along the column or row shows the highest correlation with the intensity profile of the same column or row in the stretched image.

According to Figure 7, the estimated horizontal compression for the elongation from 50 µm to 100 µm, and 100 µm to 150 µm are, respectively, 0.2% and 0.05%. The estimated vertical stretch is 0.8% and 0.44%. These compression and stretch are an order of magnitude lower than the values in Table 2 and Table 3, which indicates that the one-dimensional DIC method does not accurately evaluate the actual deformation.

**Figure 8:**
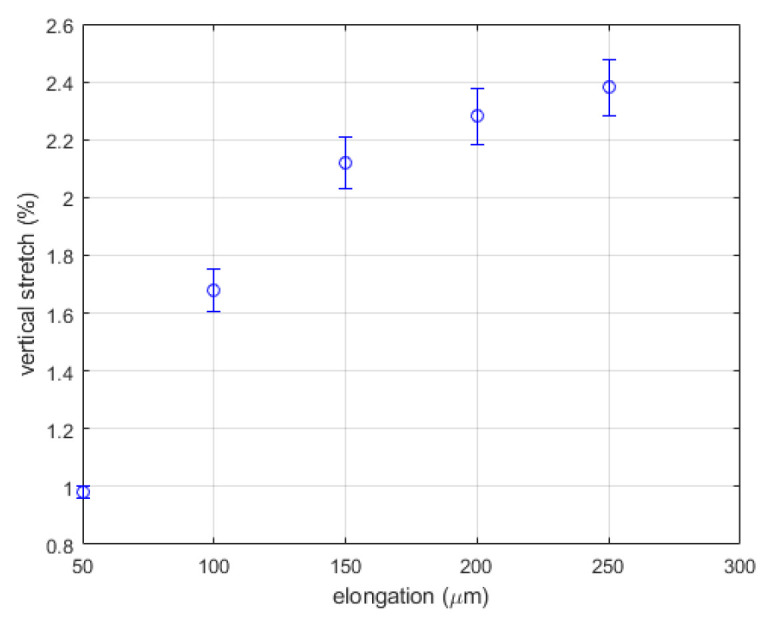
Vertical stretch evaluated from one-dimensional DIC.

Figure 8 shows the vertical stretch evaluated from the one-dimensional DIC method as a function of elongation. At elongations greater than 250 µm, the speckle fields before and after the corresponding deformation lose the correlation [24]. Consequently, the plot corresponding to Figure 7 does not show a clear peak. We speculate that when the elongation increases to 250 µm or higher, the deformation becomes significantly inhomogeneous and the stretch tends to become concentrated in a small region, reducing the overall correlation of the entire image. The nonlinear behavior observed in Figure 8 seems to result from this reduction in correlation. The increase in the error bar with the elongation supports this speculation.

These observations indicate that the intensity correlation in the space-domain is not a good method to evaluate the stretching factor for an entire column or row.

#### 3.2.2. Two-Dimensional DIC

**Figure 9:**
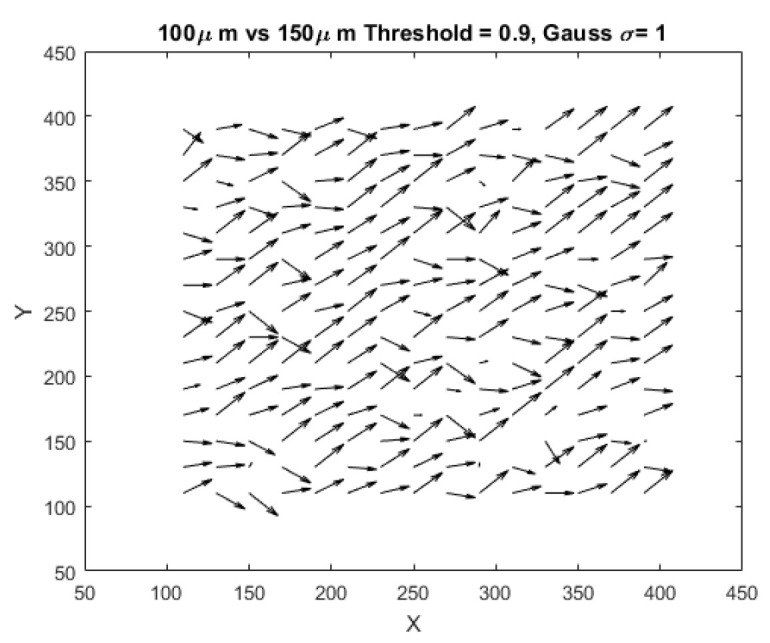
Results from two-dimensional digital image correlation method in space-domain.

Figure 9 is a quiver plot that exhibits the vector field of the displacement experienced by the specimen when the elongation increases from 100 µm to 150 µm.

It is seen that along column 200 the displacement vectors are oriented at approximately 45${}^{\circ }$. This orientation is consistent with the above observation that the magnitude of the horizontal and vertical strain is at the same level.

The differences in orientation between vectors can be attributed to a variety of factors, including error. However, preliminary results from our thermal imaging study, presented in Appendix B, indicate that the film specimen exhibits an alternating pattern of stretch and compression along the columns, implying that the observation in Figure 9 may have significance. Additional research, including more in-depth analyses in the frequency domain, is required to confirm this behavior, and will be the subject of our future work.

### 3.3. One-Dimensional Image Scaling Method in Frequency Domain

#### Image Scaling and Gaussian Filtering

**Figure 10:**
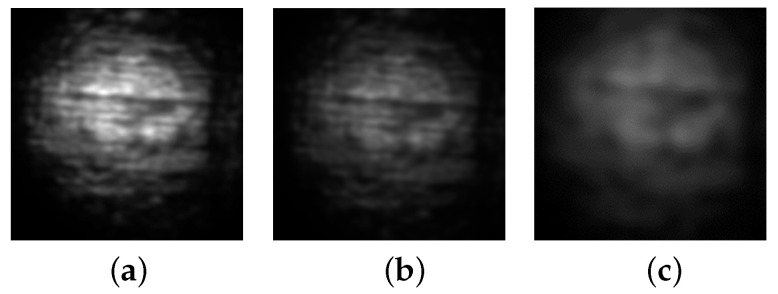
Comparison between unfiltered and Gaussian filtered images. (**a**) Unfiltered, (**b**) Filtered, σ=20, (**c**) Filtered, σ=30.

High frequency speckles (the speckle noise) superposed on the linear dark-fringes compromises the accuracy of this method. Gaussian filtering reduces the speckle noise and, therefore, is an effective way to process the image prior to applying this technique. Figure 10 shows the effect of Gaussian filtering with two different filtering parameters σ (standard deviation).

**Figure 11:**
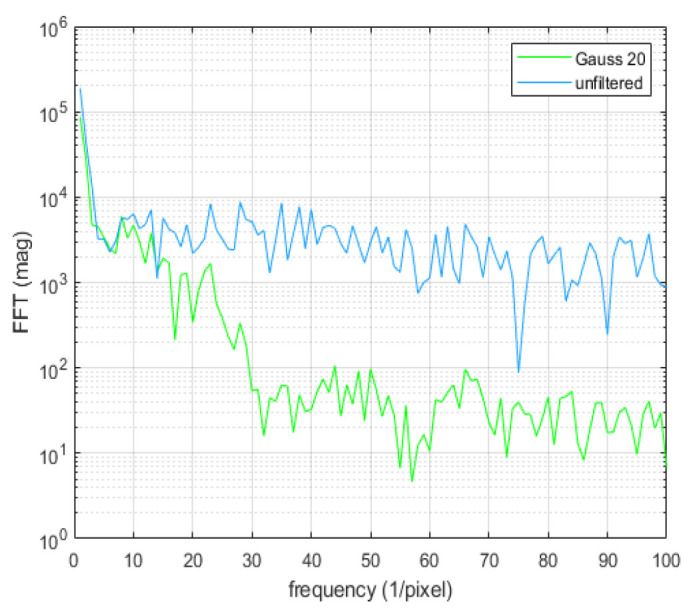
Fourier spectrum of Gauss filtered with σ=20 (pixel) and unfiltered.

Choosing a good frequency range for the analysis is not straightforward. One idea is to use the frequency range that the filtering does not alter. Figure 11 shows the Fourier spectrum along a vertical line near the horizontal center of Figure 10 (a) (unfiltered) and (b) (Gaussian-filtered with standard deviation σ of 20).

It is seen that the frequency range of 1–10 pixel${}^{-1}$ is unaffected by the filtering. Thus, the image shown in Figure 10b is used for the rest of the analysis.

According to the above argument, we use the frequency components in the range of 1–10 pixel${}^{-1}$ and discard all other frequency components including the DC (0 pixel${}^{-1}$) component as it represents the uniform background intensity. We repeat the procedure for the following seven elongation data; no elongation, elongation of 50, 100, 200, 300, 400, and 500 µm. Since the specimen stretched vertically, the Fourier spectrum compresses wias elongation increases. As the spectrum compresses on the frequency axis, the area of the spectrum decreases.

Using the analytical steps described in Section 2.3.4, the spectral compression was evaluated by computing the area of the spectrum in the frequency range of 1–10 pixel${}^{-1}$. Due to the variation of the total optical intensity between measurements for reasons mentioned in Section 2.3.4, the Fourier spectrum of the differential intensity over 1–10 pixel${}^{-1}$ is normalized to the differential intensity’s total intensity, which is the sum of gray-scale values of the numerically differentiated data.

**Figure 12:**
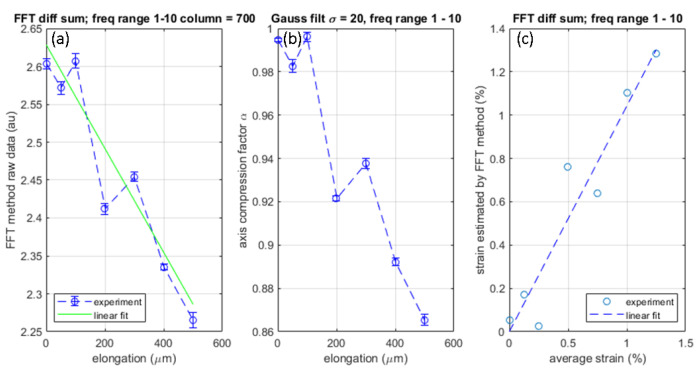
(**a**) Normalized Fourier spectrum vs. elongation; (**b**) Calibrated Fourier spectrum vs. elongation; (**c**) Comparison of stretching factor evaluated with frequency-domain image scaling method and average normal strain evaluated from elongation.

The above procedures yield Figure 12. The vertical axis of Figure 12a is the area of the normalized Fourier spectrum of the differential intensity. The solid line is the best linear fit to the data points.

Since the y-intercept of this line represents the FFT spectrum area before the specimen stretches, the division of all other data points by the y-intercept provides us with the axis compression factor α as shown in Figure 12b. The specimen’s stretching (the normal strain) ϵ is the reciprocal of α. We can find the average normal strain by dividing the elongation by the specimen’s length. Thus, Figure 12c compares the specimen’s stretching factor evaluated by the above-described FFT method (the experimental ϵ) with the average normal strain calculated from the elongation (the estimated ϵ). The dashed-line is the best linear fit. The experimental ϵ and estimated ϵ show reasonable agreement indicating a linear relation with the slope of unity.

## 4. Concluding Remarks

### Summary and Findings

In summary, this study applies DIC and speckle pattern techniques to characterize unique patterns observed in LLDPE film undergoing tensile deformation. This insight is of particular interest when considering desirable financial outcomes in industries like commercial shipping that aim to optimize time, cost, and efficiency. Images of the film’s surface undergoing deformation were projected onto a screen using a linearly polarized, collimated Helium-Neon laser beam. Resulting digital images contain speckle and diffraction-like fringe patterns and were used in both the spatial and frequency domain analyses as well. In the space domain, the random speckle pattern was used to register local displacement generated from the deformation. The fringes, however, exhibit periodic features consistent with the structural grooves due to the polymer arrangement on the film’s surface, and compromises the registration of local displacement. These periodic groove patterns are used in the frequency domain analysis. Conventional DIC in the space domain applies when deformation is uniform over the entire specimen or analysis is limited to small, localized regions. Overall, spatial DIC is found to be unreliable and inefficient for the present application. The frequency domain analysis, however, is found to be capable of revealing average global deformation under non-uniform conditions. Ultimately, we conclude that analysis in the frequency domain using the linear groove patterns is superior to the traditional methods because it is capable of revealing the global average deformation.

More quantitatively, the study has led to the following findings.

1. The space-domain one-dimensional DIC exhibits an order of magnitude smaller (a factor of two smaller at best) strain than the expected value at the 1% or lower strain level. The speckle patterns lost correlation when the strain level becomes approximately four times higher. We suspect the reason behind this finding is as follows. The speckles are formed by the diffusive nature of the transmitted light due to the randomness of the short branches that form the polymer. When the film specimen stretches these short branches shift depending on their original orientations. Therefore, the shift of the associated speckle patterns are not necessarily in line with the direction of the stretch. Hence, at a certain point of elongation, the speckle pattern starts to change randomly.
2. Frequency domain analysis appears superior to its space-domain counterparts. Unlike the speckle patterns due to the random structure of the polymer, the frequency domain analysis uses the periodic structural grooves. Consequently, the linear dark-fringes resulting from this periodic structure correlates well with the stretch of the film specimen. The random speckle patterns compromise this correlated change in the linear dark-fringes. Proper low-pass filtering diminishes this compromising effect. In the present case, Gaussian filtering with the standard deviation of 20 is found effective. With this configuration, the estimated global strain shows reasonable agreement with the expected strain level at least up to 1.4%.
3. The two-dimensional DIC based on the convolutional algorithm is found effective to some extent. However, some of the vectors in the resultant local displacement field exhibit seemingly incorrect directions. Whether these vectors are incorrectly produced by the algorithm or possibly representing the actual material’s behavior is an open question. The preliminary result of additional thermal imaging experiments indicate a similar behavior of deformation. It is the subject of our future research.

## Figures and Tables

**Table 1:** Camera specifications.

Focal Length	52 mm
Aperature	ƒ/2
Frame Rate	1/33 s

**Table 2:** Horizontal normal strain estimated from representative coordinates.

	($x_{1}$, $y_{1}$)	($x_{2}$, $y_{2}$)	$x_{2}-x_{1}$
100 µm	(178.3, 225.8)	(262.2, 164.0)	262.2 − 178.3 = 83.9
150 µm	177.0, 223.0)	259.2, 162.6)	259.2 − 177.0 = 82.2
	change in length	$\Delta (x_{2}-x_{1})$	82.2 − 83.9 = −1.7
	normal strain	$\epsilon_{xx}$	−1.7/83.9 = −0.020 = −2.0%

**Table 3:** Vertical normal strain estimated from representative coordinates.

	($x_{3}$, $y_{3}$)	($x_{4}$, $y_{4}$)	$y_{4}-y_{3}$
100 µm	(202.6, 119.1)	(193.4, 307.2)	307.2 −119.1 = 188.1
150 µm	(203.8, 112.1)	(192.9, 304.4)	304.4 − 112.1 = 192.3
	change in length	$\Delta (y_{4}-y_{3})$	192.3 − 188.1 = 4.2
	normal strain	$\epsilon_{yy}$	4.2/188.1 = 0.022 = 2.2%

**Figure A2:**
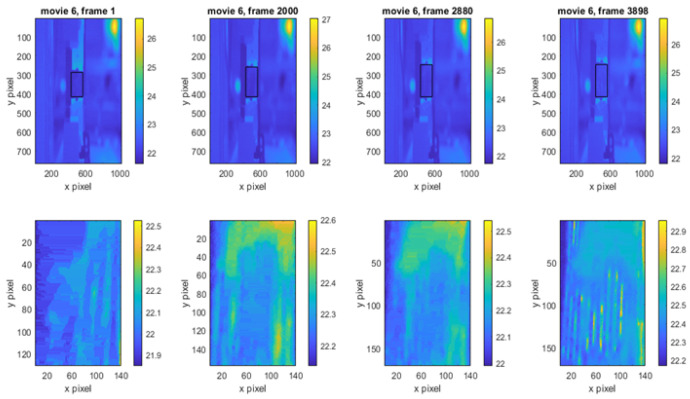
Preliminary analysis using thermal imaging.

## Data Availability

Not applicable.

## Abbreviations

The following abbreviations are used in this manuscript:

DIC	Digital Image Correlation
FFT	Fast Fourier Transform
LLDPE	Linear Low-Density Polyethylene

## Appendix A. Verification of the Fourier Scaling Property

As the LLDPE film stretches, the space between linear dark-fringes, or the spatial frequency of the fringes, changes. Section 2.3.3 describes how this study uses the Fourier scaling property (Equation (5)) for frequency-domain analysis.

This method evaluates this change in the spatial frequency in the frequency domain as follows.

Consider that f(x) represents the gray-scale of the projected image before the stretch along the *x*-axis. The Fourier transform of this function is given as follows.

(A1) $$F\left(\omega \right)=\int_{-\infty }^{\infty }f\left(x\right)e^{-j\omega x}dx$$

Stretching/compressing the material is equivalent to multiplying the coordinate axis by a factor α as ξ=αx. This multiplication alters the Fourier transform as follows.

(A2) $$F_{\alpha }\left(\omega \right)=\int_{-\infty }^{\infty }f\left(\alpha x\right)e^{-j\omega x}dx=\int_{-\infty }^{\infty }f\left(\xi \right)e^{-j\omega \frac{\xi }{\alpha }}\frac{d\xi }{\alpha }=\frac{1}{\alpha }\int_{-\infty }^{\infty }f\left(\xi \right)e^{-j\omega \frac{\xi }{\alpha }}d\xi$$

Next consider Fourier transform of g(ξ)=df(ξ)/dξ, $G_{\alpha }\left(\omega \right)$. From Equation (A2),

(A3) $$G_{\alpha }\left(\omega \right)=\frac{1}{\alpha }\int_{-\infty }^{\infty }g\left(\xi \right)e^{-j\omega \frac{\xi }{\alpha }}d\xi =\frac{1}{\alpha }\int_{-\infty }^{\infty }\frac{df(\xi )}{d\xi }e^{-j\omega \frac{\xi }{\alpha }}d\xi$$

Integrate the right-hand side of Equation (A3) by parts.

(A4) $$G_{\alpha }\left(\omega \right)=\frac{1}{\alpha }\left\{{\left[f\left(\xi \right)e^{-j\omega \frac{\xi }{\alpha }}\right]}_{-\infty }^{\infty }-\int_{-\infty }^{\infty }f\left(\xi \right)\left(\frac{d}{d\xi }e^{-j\left(\frac{\omega }{\alpha }\xi \right)}\right)d\xi \right\}$$

We can ignore the first term on the right-hand side of Equation (A4) as it represents the highest frequency components of the complex conjugate pair of the Fourier transform. The highest frequency components correspond to the pixel-to-pixel gray-scale variation. Our purpose here is to find the change in the spatial frequency of the linear dark-fringes. The interval of the linear dark-fringes involves multiple pixels. Thus, $G_{\alpha }\left(\omega \right)$ becomes as follows.

(A5) $$G_{\alpha }\left(\omega \right)=\frac{-1}{\alpha }\int_{-\infty }^{\infty }f\left(\xi \right)\left(\frac{d}{d\xi }e^{-j\left(\frac{\omega }{\alpha }\xi \right)}\right)d\xi =j\left(\frac{\omega }{\alpha }\right)\int_{-\infty }^{\infty }f\left(\xi \right)e^{-j\left(\frac{\omega }{\alpha }\xi \right)}d\xi$$

Here, we use integration by parts and drop the first term for the same reason as Equation (A4). We can interpret the right-hand side of Equation (A5) as the Fourier transform of function g(ξ), $F\left\{g(\xi )\right\}$, with the scaled frequency ω/α.

So,

(A6) $$\left|G_{\alpha }\left(\omega \right)\right|=\left|F\left\{\frac{df(\xi )}{d\xi }\right\}\right|=\left|F\left\{g(\xi )\right\}\right|=\left|F\left\{g(\alpha x)\right\}\right|=\left(\frac{\omega }{\alpha }\right)\left|F\left(\frac{\omega }{\alpha }\right)\right|$$

The equality for the last two terms of Equation (A6) can be interpreted as a result of the combination of the differentiation and scaling properties of Fourier transform [17]. Figure A1 demonstrates the relation between a function’s stretching in the space domain and the compression of the Fourier transform of the function in the frequency domain [15]. Here Figure A1a shows the plots of the original function, along with the same function stretched by factors of 10% and 20% in the space domain. Figure A1b plots the magnitude of the Fourier transforms corresponding to the three plots in (a). It is seen that the spatial stretch compresses the Fourier spectrum.

Equation (5) used in Section 2.3.3 indicates that when the specimen stretches the Fourier transform of the spatial derivative of the function that represents the spatial variation of the gray-scale level compresses in the same fashion as Figure A1. This method uses this property to estimate the stretching factor from the compression of the Fourier spectrum.

## Appendix B. Thermal Imaging

A FLIR infrared camera was used to capture these images while the specimen was deforming. Preliminary data using thermal imaging are shown below in Figure A2. The upper row presents the temperature of the specimen along with the background (laboratory air) temperature. The frame number shown above each image indicates the region enclosed by a rectangle is the film specimen. Due to the phenomenon known as the thermoelastic effect, it is expected that the film specimen increases/decreases its temperature when it is stretched/compressed. Figure A2 indicates that as the deformation develops.

The top row of images in Figure A2 shows the full image including ambient interference, while the bottom row shows the image zoomed in to the specimen (indicated above in the box). The original length of the specimen was 2.9 mm, and the final elongation was 3.6 mm. The frame numbers correspond to vertical strain of 0%, 9.95%, 14.3%, and 19.4%, respectively.

## References

[1] Ahmed M. Importance of Linear Low Density Polyethylene (LLDPE). Technologies in Industry 4.0. https://www.technologiesinindustry4.com/2021/08/importance-of-linear-low-density-polyethylene-lldpe.html.

[2] Sciammarella C.A., Sciammarella F.M. (2012). Experimental Mechanics of Solids.

[3] Leendertz J.A. (1970). Interferometric displacement measurement on scattering surfaces utilizing speckle effect. J. Phys. E.

[4] Hild F., Roux S., Hack E., Rastogi P.K. (2012). Digital Image Correlation. Optical Methods for Solid Mechanics: A Full-Field Approach.

[5] Hild F., Roux S. (2012). Comparison of Local and Global Approaches to Digital Image Correlation. Exp. Mech..

[6] Nwanoro K., Harrison P., Lennard F. (2021). Investigating the Accuracy of Digital Image Correlation in Monitoring Strain Fields Across Historical Tapestries. Strain.

[7] Brake J., Jang M., Yang C. (2016). Analyzing the relationship between decorrelation time and tissue thickness in acute rat brain slices using multispeckle diffusing wave spectroscopy. J. Opt. Soc. Am..

[8] Hajjarian Z., Nia H.T., Ahn S., Grodzinsky A.J., Jain R.K., Nadkarni S.K. (2016). Laser Speckle Rheology for evaluating the viscoelastic properties of hydrogel scaffolds. Sci. Rep..

[9] Duncan D.D., Kirkpatrick S.J., Gladish J.C. (2008). What is the proper statistical model for laser speckle flowmetry. Volume: Complex dynamics and fluctuations in biomedical photonics. Complex Dynamics and Fluctuations in Biomedical Photonics V.

[10] Popov I., Weatherbee A., Vitkin I.A. (2017). Statistical properties of dynamic speckles from flowing Brownian scatterers in the vicinity of the image plane in optical coherence tomography. Biomed. Opt. Express.

[11] Zhu Y.K., Tian G.Y., Lu R.S., Zhang H. (2011). A review of optical NDT technologies. Sensors.

[12] Quino G., Chen Y., Ramakrishnan K.R., Martínez-Hergueta F., Zumpano G., Pellegrino A., Petrinic N. (2020). Speckle patterns for DIC in challenging scenarios: Rapid application and impact endurance. Meas. Sci. Technol..

[13] Jerabek M., Major Z., Lang R.W. (2010). Strain Determination of Polymeric Materials Using Digital Image Correlation. Polym. Test..

[14] Passieux J.C., Bugarin F., David C., Périé J.N., Robert L. (2015). Multiscale Displacement Field Measurement Using Digital Image Correlation: Application to the Identification of Elastic Properties. Exp. Mech..

[15] Arai Y. (2014). Electronic speckle pattern interferometry based on spatial information using only two sheets of speckle patterns. J. Mod. Opt..

[16] Mazzoleni P., Matta F., Zappa E., Sutton M.A., Cigada A. (2015). Gaussian pre-filtering for uncertainty minimization in digital image correlation using numerically-designed speckle patterns. Opt. Lasers Eng..

[17] Ding X., Wang Z., Hu G., Liu J., Zhang K., Li H., Ratni B., Burokur S.N., Wu Q., Tan J. (2020). Metasurface holographic image projection based on mathematical properties of Fourier transform. PhotoniX.

[18] Kim J. (2018). Evaluation of resonance characteristics of thin film with improved opto acoustic method. Masters Abstracts International; Ann Arbor: ProQuest Dissertations & Theses.

[19] Yoshida S. (1999). Optical interferometric study on deformation and fracture based on physical mesomechanics. Phys. Mesomech..

[20] Takahashi S., Yoshida S., Sasaki T., Hughes T. (2021). Dynamic ESPI Evaluation of Deformation and Fracture Mechanism of 7075 Aluminum Alloy. Materials.

[21] Fransinski L.J. (2016). Covariance mapping techniques. Phys. B At. Mol. Opt. Phys..

[22] Yuan Y., Zhan Q., Xiong C., Huang J. (2017). Digital image correlation based on a fast convolution strategy. Optics Lasers Eng..

[23] Rao Y.R., Prathapani N., Nagabhooshanam E. (2014). Application of normalized cross correlation to image registration. Int. J. Res. Eng. Technol..

[24] Hoq M.E. (2020). An Investigation of Image Correlation for Real-Time of Wrapping Film Deformation. Masters Abstracts International; Ann Arbor: ProQuest Dissertations & Theses.

